# Chromatic dispersion and thermal coefficients of hygroscopic liquids: 5 glycols and glycerol

**DOI:** 10.1038/s41597-023-02819-3

**Published:** 2023-12-13

**Authors:** Daniel Jakubczyk, Gennadiy Derkachov, Kwasi Nyandey, Sima Alikhanzadeh-Arani, Anastasiya Derkachova

**Affiliations:** 1 Institute of Physics, Polish Academy of Sciences, al. Lotników 32/46, 02-668 Warsaw, Poland; 2 Laser and Fibre Optics Centre, Department of Physics, School of Physical Sciences, College of Agriculture and Natural Sciences, University of Cape Coast, Cape Coast, Ghana; 3 Present Address: Farhangian University, P.O. Box 14665-889, Tehran, Iran

**Keywords:** Characterization and analytical techniques, Characterization and analytical techniques

## Abstract

Chromatic dispersion and thermal coefficients of 6 hygroscopic liquids: ethylene glycol, diethylene glycol, triethylene glycol, tetraethylene glycol, propylene glycol (propane-1, 2-diol), and glycerol were measured in the range from 390 to 1070 nm for temperatures from 1 to 45 °C. A modified Abbe refractometer was utilised. Special care was taken to avoid contaminating the liquids under the test with water and solid particles. The measurement uncertainties were analysed. It was noticed that (in the given range and within the available measurement accuracy) the dependence of the refractive indices on the wavelength and temperature could be considered independently. Thus, thermal coefficients were found for each wavelength used, and their weak dependence on the wavelength was recognised. Then the Sellmeier equation was fitted to the experimental results for each temperature.

## Background & Summary

Achieving ultimate accuracy in optical remote sensing and particle characterisation requires accurate values of refractive indices of characterised materials and host media for a given wavelength and temperature. Since we tackle such issues in our research (e.g.^1–3^), we’ve looked for the available refractive index data and usually found it insufficient for our purposes. Thus, we decided to build a dedicated setup to measure the refractive indices of liquids as a function of both wavelength and temperature. However, since we studied popular hygroscopic liquids, the results seem to be worth sharing. A specific application that may serve as a good example, is industrial dehydration of natural gas with glycols (most prominently triethylene glycol)^4^. The amount of absorbed water could be accurately assessed by refractive index measurement of the mixture, which calls, among others, also for accurate refractive index data of the pure liquids.

Accurate measurements of refractive indices – their chromatic dispersion and temperature coefficient in particular – of hygroscopic liquids, require special care to avoid contamination with water. A glimpse into e.g. Landolt-Börnstein database^5,6^ reveals a large spread of results obtained by different authors over the past decades, which may be due precisely to pollution issues. We shall briefly discuss some of the results that can be found in the literature on the ethylene glycol (MEG) – a comparatively well investigated liquid, widely used as an engine coolant, anti-freeze, de-icing agent, polymerisation precursor and desiccant. The results we discuss are presented in Fig. 2 together with the results of our experiments. Solid black dots represent different n_D20_ measurements taken until late 1980s. It can be noticed that there are more outliers towards the lower values. The newer results of Tsierkezos^7^ or Jimenez^8^ – taken also at different temperatures (open stars and diamonds respectively), belong to the higher ones. The old (1916) but extensive results of Karvonnen on chromatic dispersion^9^ are in agreement with these. It seems to indicate that even before the molecular sieves came into everyday use, controlling the contaminating water content was possible with well-planned procedures. Our results are also in agreement with all the later mentioned. The contemporary measurements of chromatic dispersion in MEG are rather sparse^10–12^. A fairly recent work by Sani and Dell’Oro^11,12^ seems promising. An indirect method was utilised – the absorption (imaginary part of the refractive index) in MEG was measured for a very wide range of wavelengths and Kramers-Kronig relation was invoked. However, neither the temperature, at which the dispersion curve was obtained (possibly 20 °C – room temperature), nor the purity of MEG was stated. The older measurements of Voellemy^13^ at 21.9 °C and Timmermans *et al*.^14^ at 15 °C are consistent with those, while contemporary measurement of Kozma *et al*. at 22 °C clearly is not^10^.

Most of the systematic measurements versus (either) wavelength or temperature are about century old. The measurements simultaneously dependent on wavelength and temperature are rare. In this work, we present such measurements we performed for 5 commercially available glycols and glycerol. We took extreme care not to contaminate them with water beyond the amount stated by the manufacturer, though we didn’t use molecular sieves to avoid possible contamination with nanoparticles, which is crucial for our applications (light scattering). We measured dispersion of their refractive indices from 394 to 1071 nm for temperatures from 1 to 45 °C. We describe the setup and the procedures we used in detail.

## Methods

### The refractometer setup

**Fig. 1:**
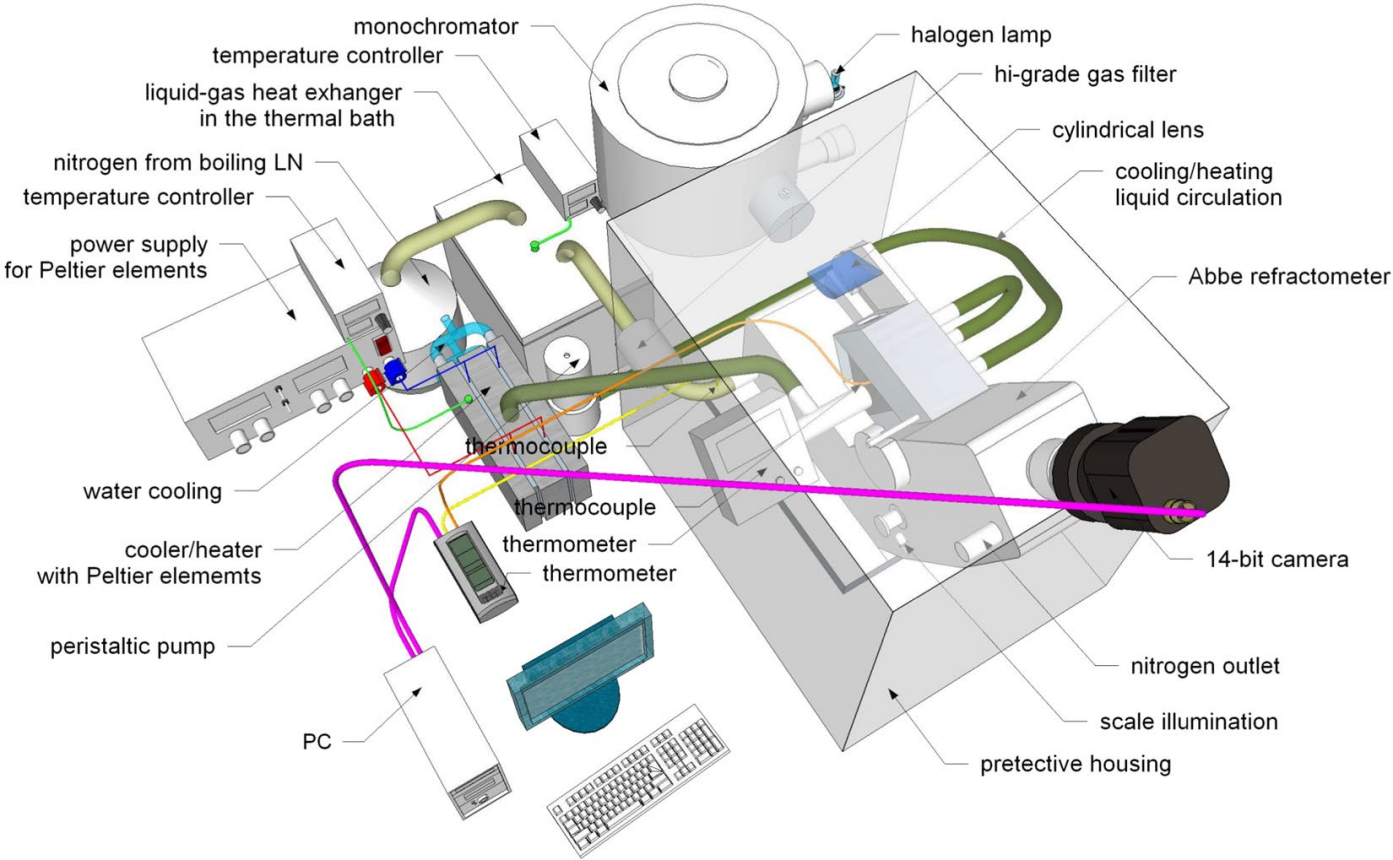
Drawing of the measurement setup. Protective housing represented schematically.

The experimental setup – see Fig. 1 – was based on a commercial Abbe refractometer (AR-4, Müller), which we modified to measure the chromatic dispersion of refractive index and its temperature dependence. We partly followed^15^ (compare also e.g.^16^). So the compensator (2 Amici prisms) was set to maximum dispersion. In consequence, the index of refraction read from the scale had to be corrected with the formula (6) from^15^, where we verified the prism material and apex angle by carefully calibrating the device with water. The light at a desired wavelength was provided through cylindrical lens from a monochromator (SPM 1, Carl Zeiss Jena^17^) with halogen lamp (H1 automotive bulb) illumination. Monochromator calibration was performed *in-situ* with a small grating spectrometer (USB4000, Ocean Optics; 1.4 nm resolution). We equipped the refractometer with a 14-bit digital camera (GC651MP, Smartek Vision) looking through the refractometer’s eyepiece with an additional camera objective (f = 6 mm, f/1.6, aberration correction including IR), so that both the bright and dark area (shadow resulting from the total internal reflection in the prism) with the crosshair superimposed and the scale were in the FoV simultaneously. In this way we could obtain sensible measurements in the spectral range from 394 to 1071 nm. The image of the bright and dark area was processed numerically using a Matlab program^18^ written in the lab (clipping, background subtraction, vertical summation, smoothing) and the shadow edge brightness profile was obtained. The derivative of the profile exhibits a minimum corresponding to the inflection point on the brightness profile. The position of the shadow edge was identified with this point. The crosshair centre was determined by pointing at it in a magnified image at the beginning of the measurement series. Thus, the measurement consisted of adjusting the profile derivative minimum to the crosshair centre. The refractometer allows for measurements at different temperatures by circulating a liquid at a desired temperature through the prisms jacket. The temperature of the circulating cooling/heating liquid was maintained with in-lab built Peltier element-controlled heat exchanger with local stabilisation loop. A separate K-type thermocouple was placed directly next to the prisms-liquid contact surface to measure the temperature of the liquid accurately. It was measured with the calibrated CHY506R electronic thermometer (CHY Firemate Co.). Dry, filtered N_2_ gas, obtained from liquid nitrogen, was flowed through the refractometer chassis and into the (plastic) protective housing in which the device was kept. The temperature of N_2_ gas was stabilized by passing it through gas-liquid heat exchanger in a thermal bath stabilised down to ± 0.2 °C. Depending on the temperature of the refractometer prisms, the temperature of the bath was set between −5 and 0 °C to keep it significantly lower than that of the prisms. The pipe leading the gas to the refractometer was thermally insulated, but the heat transfer was found significant there. Thus, the N_2_ temperature was measured continuously at the entrance of the refractometer to ensure that during the experimental run it was stable down to ± 1 °C. Depending on the temperature of the bath, it stabilised in the 1–6 °C range. In this way, we (i) stabilized the temperature of the refractometer body, which enabled good calibration of the device; (ii) prevented condensation of water vapour (as well as other vapours) on the optical surfaces in the device; (iii) minimized diffusion of atmospheric water into the measured liquid. The relative humidity (RH) measured in the enclosure was always below 24% and the contact area of the sample in between the prisms with the enclosure atmosphere was ~10 mm^2^ (in comparison to ~10^3^ mm^2^ in contact with glass). It is difficult to obtain a very low humidity in a fairly large plastic housing, because of relatively high residual water content in plastics (see e.g.^19,20^:). However, it was confirmed with a long-time measurement that the refractive index of a sample sitting between the prisms remains constant for several hours. An ample time (both for calibration and actual measurement) was always allowed for the temperature of the device to stabilise, where the primary condition was the temporal stability of the observed shadow line.

### Measurement procedures and materials

Since for water there exist widely recognised systematic measurements of spectral dispersion versus temperature^21^, the device was calibrated with distilled water (in-lab produced) at a given temperature, before every measurement series. This fundamentally precluded calibration below 0 °C. Furthermore, in the case of water, for T→0 °C, dn/dT→0, which means that the calibration accuracy diminishes significantly towards 0 °C. Thus, for measurements at 1 °C the calibration was later augmented with data for MEG we obtained. On the other hand, at elevated temperatures, the water between the prisms dried out faster, making calibration increasingly difficult. So, ~0.5 ml (an ample amount) of distilled water was slowly (to avoid forming bubbles) poured on the surface of the prism with a clean disposable syringe. The packages with disposable syringes were kept under vacuum to dry the syringe plastic as far as possible. The excess of water was squeezed out and removed on closing the top prism. The calibration was performed first of all at 589 nm (sodium D-line) and verified at 394 and 1071 nm. After the calibration, water was removed and the surface of prisms was carefully cleaned with a soft tissue and propanol. Then, the prisms were further dried with strong nitrogen gas flow (compressed N_2_ of purity better than 99.8%). Extreme care must be taken, because the amount of liquid used for measurements is minute and even a small addition of water (or other substances) affects the measurements. After the drying, ~0.5 ml of desired liquid was poured with a new disposable syringe as it was done with water and a series of measurements were taken at a desired temperature starting from the infrared towards the ultraviolet. Since the measurement series takes about 30 min, after the end, the measurements for the longest IR wavelengths were repeated, to ensure that there was no change in experimental conditions, e.g. due to mechanical creep in the apparatus. Furthermore, after the measurement of the sample, the prisms were cleaned and the calibration with water was rechecked, in order to exclude any systematic errors introduced by the creep in the apparatus taking place during the measurement of the sample. The unsealed bottles with the hygroscopic liquids were loosely recapped and stored under vacuum to ensure that they don’t absorb atmospheric water. The lab was air-conditioned and the temperature of 22 ± 1 °C was maintained, also to keep the humidity in the lab relatively low (~45%). However there was no stabilisation of humidity in the lab, and we observed some variation versus the season of the year. The measurement series for each temperature, were repeated several times, the outlying series were discarded and the remaining were averaged. The sampled liquids are indicated in Table 1.

**Table 1:** Sampled liquids.

IUPAC name (other name)	abbrev.	CAS number	origin	purity	lot #
ethylene glycol	MEG	107-21-1	Sigma-Aldrich	anhydrous, 99.8%	STBG3967V
2, 2′-oxydiethanol (diethylene glycol)	DEG	111-46-6	Sigma	≥99.0%	BCBT4833
2, 2′-[ethane-1,2-diylbis(oxy)]di(ethan-1-ol) (triethylene glycol)	TEG	112-27-6	Alfa Aesar	99%	10198029
2-[2-[2-(2-hydroxyethoxy)ethoxy]ethoxy]ethanol (tetraethylene glycol)	TetEG	112-60-7	Alfa Aesar	99%	M17E015
propane-1, 2-diol (propylene glycol)	PG	57-55-6	Sigma-Aldrich	≥99.5%	MKC80613V
propane-1, 2, 3-triol (glycerol)		56-81-5	Sigma	BioUltra, anhydrous,≥99.5%	BCBS7814V

### Data processing and accuracy considerations

**Fig. 2:**
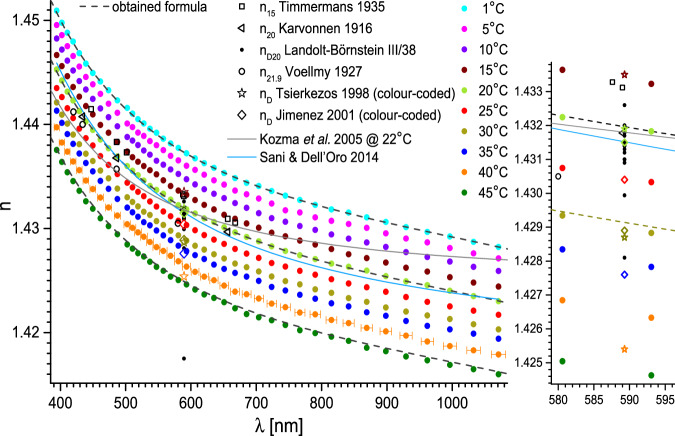
Refractive index of ethylene glycol versus wavelength and temperature. Dashed lines represent fits with the obtained formula (Eqs. (1–3)) with parameters from Tables 2, 3. The uncertainty of the wavelength is shown for clarity only for 40 °C in left panel. Essential data from Landolt-Börnstein database is also presented as: black squares^14^, triangles^9^, dots^5^, circles^13^, stars^7^ and diamonds^8^. Stars and diamonds are additionally colour-coded according to coding of our data. Two newer results are shown as grey^10^ and blue^11,12^ lines. The most significant data is referenced directly. Right panel: magnification of the region around the sodium D-line – most of the literature data falls in this region.

**Fig. 3:**
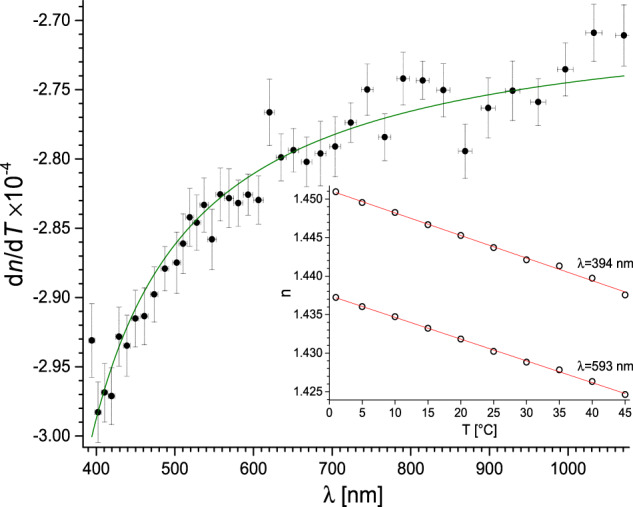
Black solid circles: d*n*/d*T*(*λ*) for MEG obtained from linear *n*(*T*) fits for each experimental *λ* point. Vertical error bars correspond to d*n*/d*T* standard error and the horizontal – to the estimated uncertainty of *λ*. Green solid line: rational function fit – Eq. 2. Inset: open circles: *n*(*T*); red solid lines: two linear fits at different *λ* (central and peripheral).

After gathering the dataset *n*(*λ, T*) – refractive indices for a set of (vacuum) wavelengths *λ* and temperatures *T* in the full available range (compare Fig. 2) – it was found that the dependence of n on *λ* and *T* (in °C) could be decomposed for each liquid under study, with *n*(*T,λ*=const) considered linear within our measurement accuracy (see Fig. 3):

1 $$n\left(\lambda ,T\right)=n\left(\lambda ,20\right)+\frac{{\rm{d}}n\left(\lambda ,T\right)}{{\rm{d}}T}\left(T-20\right).$$

**Fig. 4:**
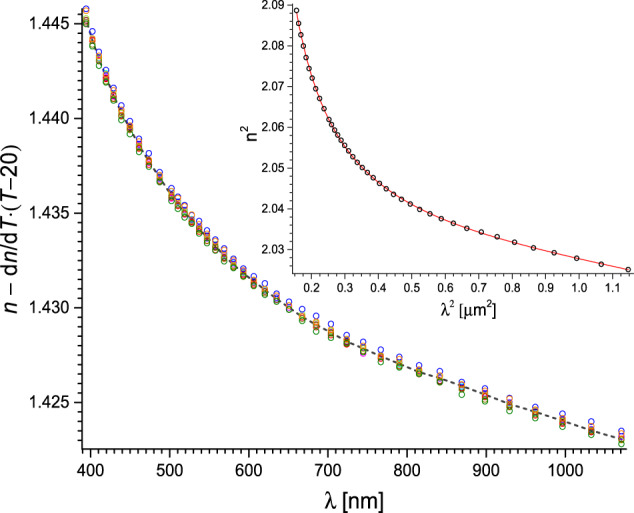
The *n*(*λ*) traces for all temperatures for MEG were shifted to overlap the trace corresponding to 20 °C. The black dashed line shows the median of shifted traces. Inset: Sellmeier equation fitted (solid red line) to the obtained median points (black open circles).

Thus, d*n*/d*T* was found from a linear fit for each experimental *λ* point. The mean relative standard error of d*n*/d*T* is below 1% for all studied liquids. In inset in Fig. 3, two such fits at different *λ* (central and peripheral) for MEG are presented. Followingly, in Fig. 3 itself, we present d*n*/d*T*(*λ*) for MEG with the vertical error bars corresponding to d*n*/d*T* standard error and the horizontal – to the estimated uncertainty of *λ*. Interestingly, d*n*/d*T* displays a (weak) non-linear dependence on *λ*. A rational function, which is in line with the Sellmeier equation, was found to fit very well (COD = 0.94 for MEG):

2 $$\frac{{\rm{d}}n\left(\lambda ,T\right)}{{\rm{d}}T}={A}_{{\rm{T}}}+\frac{{B}_{{\rm{T}}}}{\lambda -{C}_{{\rm{T}}}}{\rm{,}}$$

whee *A*_T_ constant is associated mainly with thermal expansivity (density change) of the liquid and *B*_T_ and *C*_T_ parameters have similar sense as in Sellmeier equation (see below). Then, using Eq. 1, *n*(*λ*) traces for all temperatures were shifted to overlap the trace corresponding to 20 °C (compare Fig. 4). Median of all *n* for each *λ* was found and a two-pole Sellmeier equation

3 $${n}^{2}\left(\lambda ,20\right)=A+\frac{{B}_{{\rm{IR}}}{\lambda }^{2}}{{\lambda }^{2}-{C}_{{\rm{IR}}}}+\frac{{B}_{{\rm{UV}}}{\lambda }^{2}}{{\lambda }^{2}-{C}_{{\rm{UV}}}}$$

was fitted, where *A* accounts for the short-wavelength absorption contributions to *n* at longer wavelengths, while *B*_IR_ and *B*_UV_ are absorption resonance strengths at wavelengths ${C}_{{\rm{IR}}}^{1/2}$ and ${C}_{{\rm{UV}}}^{1/2}$ respectively. The procedure was repeated for all studied liquids.

## Data Records

All the obtained datasets (tables) are stored in Mendeley Data repository^22^. The dataset consists of tables of refractive index values versus wavelength and temperature *n*(*λ, T*) for 6 hygroscopic organic liquids^22^. Each table corresponds to one liquid:

- ethylene glycol (File n(T,lambda)_EG.csv),
- diethylene glycol (File n(T,lambda)_DEG.csv),
- triethylene glycol (File n(T,lambda)_TEG.csv),
- tetraethylene glycol (File n(T,lambda)_TetEG.csv),
- propylene glycol (propane-1, 2-diol) (File n(T,lambda)_PG.csv),
- glycerol (File n(T,lambda)_Glycerol.csv).

The measurements cover the range from 394 to 1071 nm in wavelength and from 1 to 45 °C in temperature. Each data point corresponds to a mean value of several measurements. The outlaying measurement series were discarded. The accuracy of the refractive index measurement is estimated to be ±0.0003, while the accuracy of the wavelength measurement is ~1%, and the accuracy of the temperature measurement is −0.3/ + 0.6 K.

**Fig. 5:**
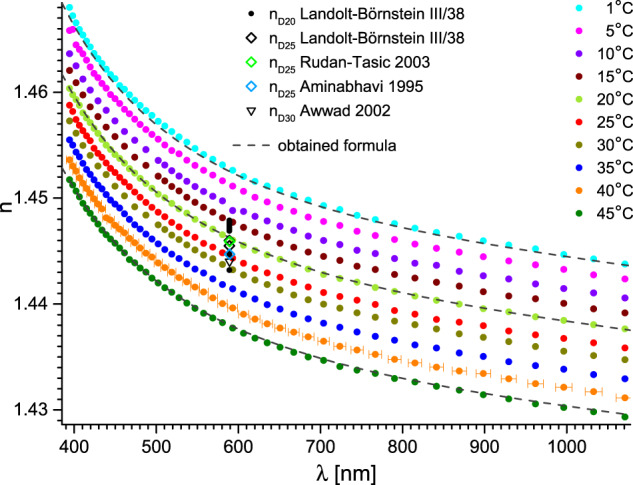
Refractive index of diethylene glycol versus wavelength and temperature. Dashed lines represent fits with the obtained formula (Eqs. (1–3)) with parameters from Tables 2, 3. The uncertainty of the wavelength is shown for clarity only for 40 °C. Essential data from Landolt-Börnstein database is also presented as: black dots^5^, black^5^, green^26^ and blue^27^ diamonds and the black triangle^28^. The most significant data is referenced directly.

**Fig. 6:**
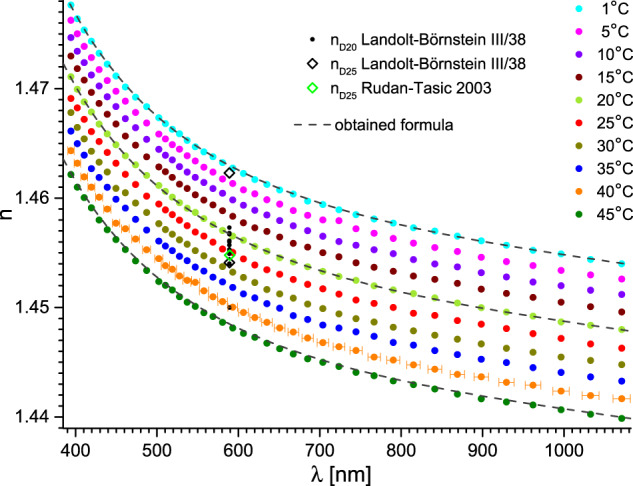
Refractive index of triethylene glycol versus wavelength and temperature. Dashed lines represent fits with the obtained formula (Eqs. (1–3)) with parameters from Tables 2, 3. The uncertainty of the wavelength is shown for clarity only for 40 °C. Essential data from Landolt-Börnstein database is also presented as: black dots^5^ and black^5^ and green^26^ diamonds. The most significant data is referenced directly.

**Fig. 7:**
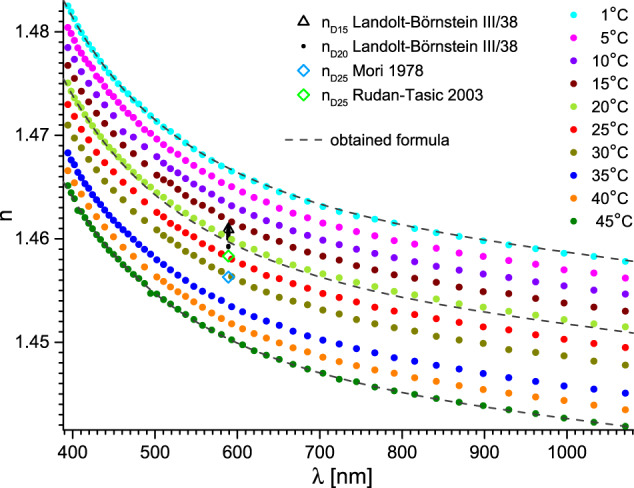
Refractive index of tetraethylene glycol versus wavelength and temperature. Dashed lines represent fits with the obtained formula (Eqs. (1–3)) with parameters from Tables 1, 2. The uncertainty of the wavelength is shown for clarity only for 40 °C. Essential data from Landolt-Börnstein database is also presented as: black triangles^5^ and dots^5^, and green^29^ and blue^26^ diamonds. The most significant data is referenced directly.

**Fig. 8:**
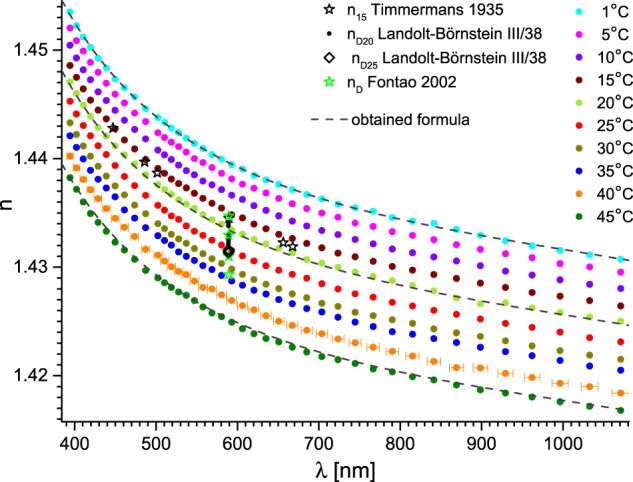
Refractive index of propylene glycol versus wavelength and temperature. Dashed lines represent fits with the obtained formula (Eqs. (1–3)) with parameters from Tables 1, 2. The uncertainty of the wavelength is shown for clarity only for 40 °C. Essential data from Landolt-Börnstein database is also presented as: black dots^5^ and diamonds^5^, and black^14^ and green stars^30^. The most significant data is referenced directly.

**Fig. 9:**
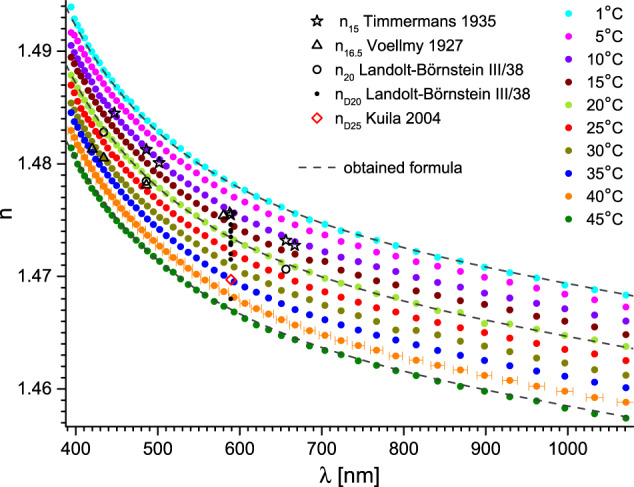
Refractive index of glycerol versus wavelength and temperature. Dashed lines represent fits with the obtained formula (Eqs. (1–3)) with parameters from Tables 1, 2. The uncertainty of the wavelength is shown for clarity only for 40 °C. Essential data from Landolt-Börnstein database is also presented as: black stars^14^, triangles^13^, circles^5^, dots^5^ and the red diamond^31^. The most significant data is referenced directly.

The obtained Sellmeier equation coefficients and thermal coefficients, for *λ* expressed in μm, are presented in Tables 2, 3 respectively. In Figs. 5–9 we also present corresponding *n*(*λ*,*T*) graphs for all studied liquids, comprising also relevant data from the literature. In case of less popular liquids, the chromatic dispersion data was not available for comparison.

**Table 2:** Sellmeier equation coefficients for *λ* in μm, found from the presented experiments.

liquid	Sellmeier equation coefficients^a^				
	*A*	*B*_IR_	*C*_IR_	*B*_UV_	*C*_UV_
ethylene glycol	1.34238 ± 3 × 10^−5^	0.0137 ± 1 × 10^−4^	3.12 ± 0.02	0.68263 ± 3 × 10^−5^	0.01332 ± 1 × 10^−5^
diethylene glycol	1.60169 ± 3 × 10^−5^	0.0283 ± 3 × 10^−4^	6.08 ± 0.05	0.46338 ± 3 × 10^−5^	0.02 ± 2 × 10^−5^
triethylene glycol	1.12259 ± 3 × 10^−5^	0.00476 ± 8 × 10^−5^	2.39 ± 0.03	0.96913 ± 3 × 10^−5^	0.0108 ± 8 × 10^−6^
tetraethylene glycol	1.48424 ± 3 × 10^−5^	0.00354 ± 8 × 10^−5^	2.25 ± 0.03	0.61562 ± 3 × 10^−5^	0.01689 ± 1 × 10^−5^
propylene glycol	1.15131 ± 3 × 10^−5^	0.0082 ± 1 × 10^−4^	2.75 ± 0.02	0.87613 ± 3 × 10^−5^	0.01092 ± 8 × 10^−6^
glycerol	1.6062 ± 3 × 10^−5^	0.0622 ± 4 × 10^−4^	7.99 ± 0.05	0.53803 ± 3 × 10^−5^	0.0181 ± 1 × 10^−5^

^a^Uncertainties represent standard errors.

**Table 3:** Thermal coefficients for *λ* in μm found from the presented experiments.

liquid	thermal coefficients^a^		
	A_T_	B_T_	C_T_
ethylene glycol	−2.643 × 10^−4^ ± 3 × 10^−7^	−7.5 × 10^−6^ ± 1 × 10^−7^	0.17 ± 0.01
diethylene glycol	−3.132 × 10^−4^ ± 2 × 10^−7^	−5.61 × 10^−6^ ± 6 × 10^−8^	0.245 ± 0.011
triethylene glycol	−3.122 × 10^−4^ ± 3 × 10^17^	−6.3 × 10^−6^ ± 2 × 10^−7^	0.22 ± 0.03
tetraethylene glycol	−3.570 × 10^−4^ ± 2 × 10^−7^	−6.51 × 10^−6^ ± 6 × 10^−8^	0.235 ± 0.009
propylene glycol	−3.027 × 10^−4^ ± 3 × 10^−7^	−1.2 × 10^−6^ ± 2 × 10^−7^	0.72 ± 0.01
glycerol	−2.395 × 10^−4^ ± 5 × 10^−7^	−6.2 × 10^−6^ ± 2 × 10^−7^	0.18 ± 0.01

^a^Uncertainties represent standard errors.

## Technical Validation

### Error analysis

All the investigated samples were clear and colourless liquids, which indicates no significant absorption of light in the visible range. The literature data – where it is available (MEG^11,12^, PG^23^, glycerol^24^) – shows the absorption coefficient, in the range accessed in our experiment, to be at most of the order of 10^−6^. In consequence, the absorption of light in the samples has no impact on accuracy considerations.

The absolute precision of refractive index measurements in our setup (AR-4 refractometer equipped with additional lenses and a digital camera) was estimated as ± 1 × 10^−4^. The accuracy however was further influenced by mechanical hysteresis of the apparatus and its long term (mainly thermal) stability and was estimated as ± 3 × 10^−4^. So the vertical error bars in *n*(*λ,T*) figures are of the size of the symbols – circles.

The maximal error of the wavelength determination was associated with the accuracy of monochromator-halogen lamp system calibration. The precision of wavelength setting is better than 1.5 nm. However, in order to achieve adequately bright illumination the slits were fairly wide-open which resulted in spectrally non-uniform illumination. Spectral profiles obtained with a multimode fibre with NA = 0.22 (P600-1-SR, Ocean Optics) were not wider than 14 nm HWHM at 1071 nm and 3 nm HWHM at 394 nm. This led to the total calibration uncertainty of ~1%. The error bars for wavelength are shown for the trace corresponding to 40 °C as an example. Due to the character of the dispersion curve, the influence of these errors on the fitted Sellmeier curve is comparable to that introduced by refractive index uncertainty.

The accuracy of the calibrated CHY506R electronic thermometer with K-type thermocouple – traced to temperature standard – was estimated as ± 0.2 K. Since a significant (up to 2 K) temperature difference between prisms-liquid contact surface and the circulating liquid was observed, the temperature gradient across the prisms surface was checked with a thin calibrated (T-type) thermocouple (TT-T-40-SLE, by Omega, connected to CHY506R). It was found not greater than 0.4 K, for highest temperature gradients in the setup (45 °C at the prisms, 2 °C at N_2_ inlet to the refractometer, 22 °C ambient in the lab). So, finally, the accuracy of temperature measurements can be estimated as −0.3/ + 0.6 K. Again, the horizontal error bars in Fig. d*n*/d*T*(*λ*) (inset) would be of the size of the symbols.

As mentioned above, a significant error can be introduced into the measurement, if a hygroscopic sample is exposed to an ambient (humid) atmosphere, especially for longer periods. For instance, at 24% RH (as in the setup enclosure) the equilibrium water content in MEG is ~9 wt% (see Fig. 18 in^25^). This would lead to a decrease of refractive index by ~0.01 (compare formula at Fig. 14 in^25^). It corresponds to 10% of whole difference between refractive indices of MEG and water (*n*_MEG_ - *n*_H2O_). Similarly, the residual uncertainty of the refractive index due to the contamination with water for the MEG lot that we used (see section “The refractometer setup”) could be estimated as ~2 × 10^−4^ (0.2% of *n*_MEG_ - *n*_H2O_), so the corresponding vertical error bars in *n*(*λ*, *T*) figures would be below the size of the symbols – circles. In view of the above analysis, extreme care was taken to a avoid any prolonged contact of samples with the atmosphere or water-saturated containers – syringes, and the possible water intake during the experiment was carefully monitored as described in the previous sections.

As explained above, the measurements, in which the systematic errors were spotted by recalibration of the setup with water, were simply discarded.

The standard errors of Sellmeier equation coefficients and thermal coefficients (Tables 2, 3) are reported by the fitting procedure (simplex algorithm). Since, from the point of view of the optimization algorithm, both equations are over-parameterized, the errors tend to be significant, and the exact physical meaning of the values obtained is somewhat questionable. However, they are quite satisfactory from an engineering point of view.

Graphical comparison of the obtained results with literature data

## Data Availability

The custom Matlab code used for experiment control and data collection is specific to our setup, as described above in section “The refractometer setup”. It was running in Matlab version 2015b, 64 bit. It is stored publically in GitHub repository^18^.

## References

[1] Hołyst R (2013). Evaporation of freely suspended single droplets: experimental, theoretical and computational simulations. Reports Prog. Phys..

[2] Kolwas M, Jakubczyk D, Do Duc T, Archer J (2019). Evaporation of a free microdroplet of a binary mixture of liquids with different volatilities. Soft Matter.

[3] Derkachov G, Jakubczyk D, Woźniak M, Archer J, Kolwas M (2014). High-precision temperature determination of evaporating light-absorbing and non-light-absorbing droplets. J. Phys. Chem. B.

[4] Cao S (2018). Mass Transfer Study of Dehydration by Triethylene Glycol in Rotating Packed Bed for Natural Gas Processing. Ind. Eng. Chem. Res..

[5] Wohlfarth, C. & Wohlfarth, B. *Refractive Indices of Organic Liquids*. *Refractive Indices of Organic Liquids* vol. 38B (Springer-Verlag, 1996).

[6] Wohlfarth, C. *Optical Constants · Refractive Indices of Pure Liquids and Binary Liquid Mixtures (Supplement to III/38)*. **vol. 47** (Springer Berlin Heidelberg, 2008).

[7] Tsierkezos NG, Molinou IE (1998). Thermodynamic properties of water + ethylene glycol at 283.15, 293.15, 303.15, and 313.15 K. J. Chem. Eng. Data.

[8] Jiménez, E., Cabanas, M., Segade, L., Garc, S. & Casas, H. Excess volume, changes of refractive index and surface tension of binary 1, 2-ethanediol + 1-propanol or 1-butanol mixtures at several temperatures. **180**, 151–164 (2001).

[9] Karvonen, A. *Ann. Acad. Sci. Fenn. Ser. A* **10**(10), 1 (1916).

[10] Kozma IZ, Krok P, Riedle E (2005). Direct measurement of the group-velocity mismatch and derivation of the refractive-index dispersion for a variety of solvents in the ultraviolet. J. Opt. Soc. Am. B.

[11] Sani E, Dell’Oro A (2014). Optical constants of ethylene glycol over an extremely wide spectral range. Opt. Mater. (Amst)..

[12] Sani E, Dell’oro A (2015). Erratum: Optical constants of ethylene glycol over an extremely wide spectral range (Optical Materials (2014) 37 (36–41)). Opt. Mater. (Amst)..

[13] Voellmy H (1927). Über die Dispersion ultravioletter Strahlen durch flüssige organische Substanzen. Zeitschrift für Phys. Chemie.

[14] Timmermans MJ, Hennaut-Roland M (1935). Etude des constantes physiques de vingt composés organiques. J. Chim. Phys.

[15] Kedenburg S, Vieweg M, Gissibl T, Giessen H (2012). Linear refractive index and absorption measurements of nonlinear optical liquids in the visible and near-infrared spectral region. Opt. Mater. Express.

[16] Rheims J, Köser J, Wriedt T (1997). Refractive-index measurements in the near-IR using an Abbe refractometer. Meas. Sci. Technol..

[17] Schiek O, Winter E (1965). Two New Mirror Monochromators. Appl. Opt..

[18] Derkachov, G., Nyandey, K., Jakubczyk, D. & Derkachova, A. Refractometer_setup. https://github.com/djbyways/Refractometer_setup (2023). 10.1038/s41597-023-02819-3 PMC10719242 38092804

[19] International Organization for Standardization. ISO 62. Plastics — Determination of water absorption (2008).

[20] Duncan, B. C. & Broughton, W. R. Absorption and Diffusion of Moisture In Polymeric Materials. *Measurement Good Practice Guide* (2007).

[21] Harvey AH, Gallagher JS, Sengers JMHL (1998). Revised Formulation for the Refractive Index of Water and Steam as a Function of Wavelength, Temperature and Density. Journal of Physical and Chemical Reference Data.

[22] Jakubczyk D, Derkachov G, Nyandey K, Alikhanzadeh-Arani S, Derkachova A (2023). Mendeley Data.

[23] Otanicar TP, Phelan PE, Golden JS (2009). Optical properties of liquids for direct absorption solar thermal energy systems. Sol. Energy.

[24] Wang K (2017). Order-of-magnitude multiphoton signal enhancement based on characterization of absorption spectra of immersion oils at the 1700-nm window. Opt. Express.

[25] The MEGlobal Group of Companies. Ethylene Glycol Product Guide. 1–33 (2008).

[26] Rudan-Tasic D, Klofutar C (2003). Apparent Molar Volume and Apparent Molar Refraction of Mono-, Di-, Tri-, and Tetra(oxyethylene) Glycol in Aqueous, 1,4-Dioxane, and Benzene Solutions at 298.15 K. Monatshefte fur Chemie.

[27] Aminabhavi TM, Gopalakrishna B (1995). Density, Viscosity, Refractive Index, and Speed of Sound in Aqueous Mixtures of N,N-Dimethylformamide, Dimethyl Sulfoxide, N,N-Dimethylacetamide, Acetonitrile, Ethylene Glycol, Diethylene Glycol, 1,4-Dioxane, Tetrahydrofuran, 2-Methoxyethanol, and 2-Ethox. J. Chem. Eng. Data.

[28] Awwad AM, Al-Dujaili AH, Salman HE (2002). Relative permittivities, densities, and refractive indices of the binary mixtures of sulfolane with ethylene glycol, diethylene glycol, and poly(ethylene glycol) at 303.15 K. J. Chem. Eng. Data.

[29] Mori S (1978). Response correction of differential refractometer for polyethylene glycols in size exclusion chromatography. Anal. Chem..

[30] Fontao MJ, Iglesias M (2002). Effect of Temperature on the Refractive Index of Aliphatic Hydroxilic Mixtures (C2-C3). Int. J. Thermophys..

[31] Kuila DK, Lahiri SC (2004). Comparison of the macroscopic molecular properties in understanding the structural aspects of mixed aquo-organic binary mixtures. Zeitschrift fur Phys. Chemie.

