# Author Correction: Chromatic dispersion and thermal coefficients of hygroscopic liquids: 5 glycols and glycerol

**DOI:** 10.1038/s41597-024-03547-y

**Published:** 2024-07-03

**Authors:** Daniel Jakubczyk, Gennadiy Derkachov, Kwasi Nyandey, Sima Alikhanzadeh-Arani, Anastasiya Derkachova

**Affiliations:** 1 Institute of Physics, Polish Academy of Sciences, al. Lotników 32/46, 02-668 Warsaw, Poland; 2 Laser and Fibre Optics Centre, Department of Physics, School of Physical Sciences, College of Agriculture and Natural Sciences, University of Cape Coast, Cape Coast, Ghana; 3 Present Address: Farhangian University, P.O. Box 14665-889, Tehran, Iran

**Keywords:** Characterization and analytical techniques, Characterization and analytical techniques, Characterization and analytical techniques, Characterization and analytical techniques

Correction to: *Scientific Data* 10.1038/s41597-023-02819-3, published online 13 December 2023

The original versions of Figure 2 and Table 2 were incorrect, taken from an earlier fit of the data that was inconsistent with figures 5-9. Uncertainties presented in Table 2 were also calculated incorrectly and displayed too few significant figures. Both elements (Table 2 and Figure 2) have been updated in the PDF and HTML version of the paper. The current and previous versions of these tables are included here for transparency.

Previous Figure 2
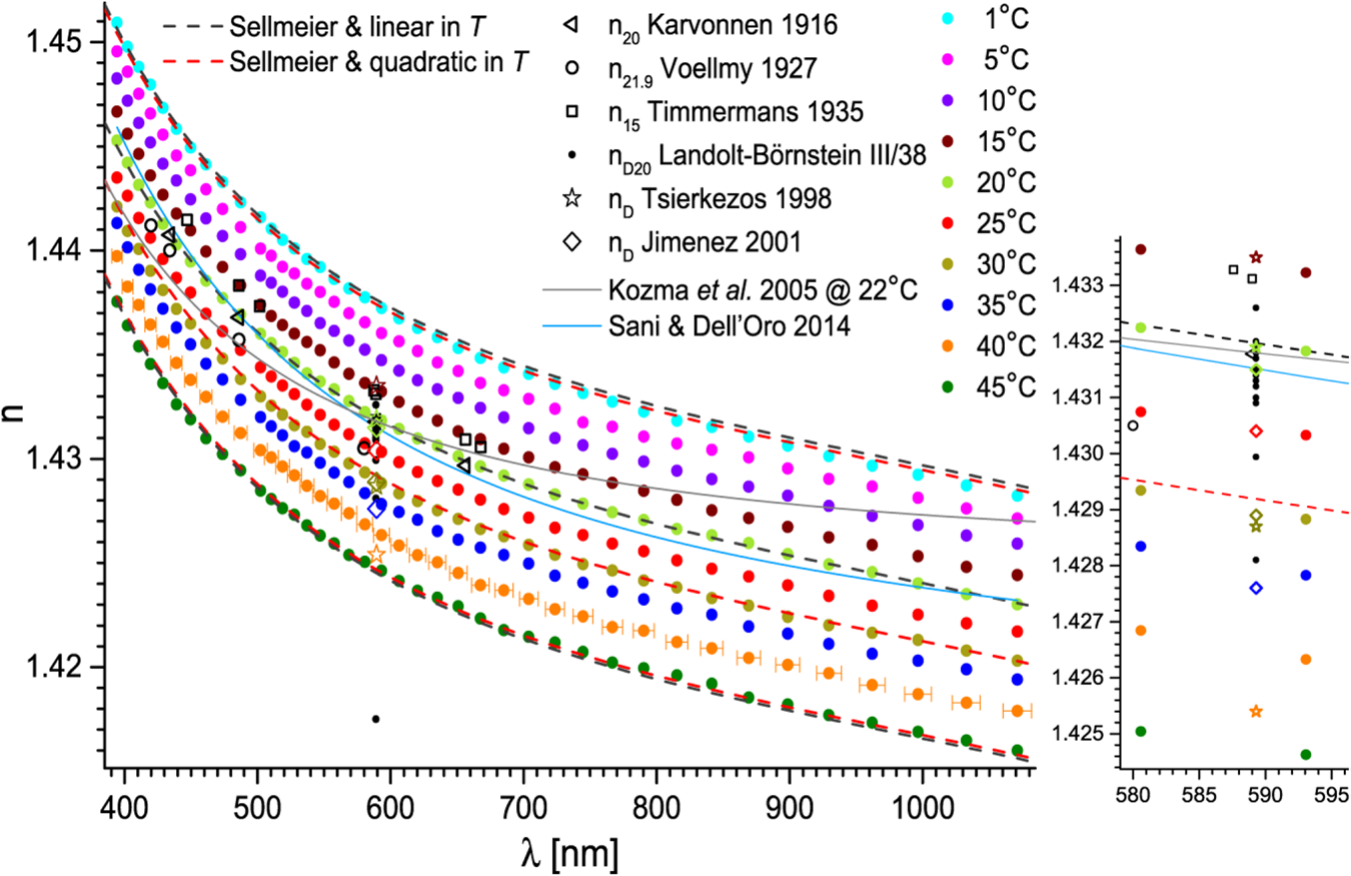


New Figure 2
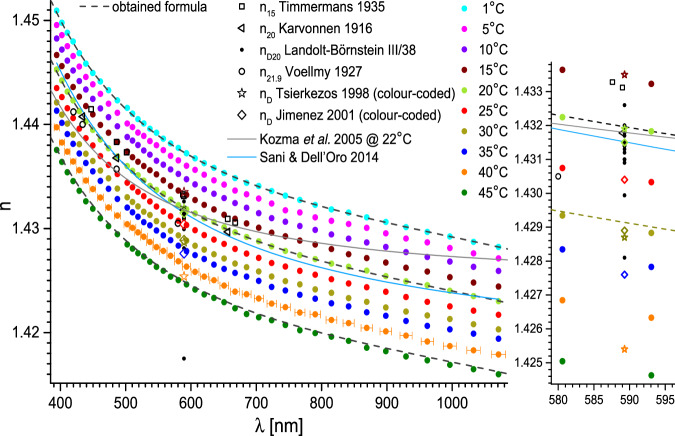


Previous Table 2

**Table:** 

liquid	Sellmeier equation coefficients^a^				
	*A*	*B*_IR_	*C*_IR_	*B*_UV_	*C*_UV_
ethylene glycol	0.6 ± 0.8	0.0076 ± 0.0051	2.5 ± 0.7	1.4 ± 0.8	0.007 ± 0.004
diethylene glycol	1.6 ± 0.1	0.03 ± 0.05	6 ± 7	0.5 ± 0.1	0.02 ± 0.003
triethylene glycol	1.24 ± 0.25	0.008 ± 0.009	3.1 ± 1.8	0.85 ± 0.25	0.012 ± 0.003
tetraethylene glycol	1.48 ± 0.11	0.0035 ± 0.0032	2.25 ± 0.81	0.62 ± 0.11	0.0169 ± 0.0025
propylene glycol	1.15 ± 0.28	0.008 ± 0.006	2.75 ± 0.95	0.9 ± 0.3	0.011 ± 0.003
glycerol	1.61 ± 0.12	0.06 ± 0.09	8 ± 9	0.54 ± 0.12	0.018 ± 0.003

New Table 2

**Table:** 

liquid	Sellmeier equation coefficients^a^				
	*A*	*B*_IR_	*C*_IR_	*B*_UV_	*C*_UV_
ethylene glycol	1.34238 ± 3 × 10^−5^	0.0137 ± 1 × 10^−4^	3.12 ± 0.02	0.68263 ± 3 × 10^−5^	0.01332 ± 1 × 10^−5^
diethylene glycol	1.60169 ± 3 × 10^−5^	0.0283 ± 3 × 10^−4^	6.08 ± 0.05	0.46338 ± 3 × 10^−5^	0.02 ± 2 × 10^−5^
triethylene glycol	1.12259 ± 3 × 10^−5^	0.00476 ± 8 × 10^−5^	2.39 ± 0.03	0.96913 ± 3 × 10^−5^	0.0108 ± 8 × 10^−6^
tetraethylene glycol	1.48424 ± 3 × 10^−5^	0.00354 ± 8 × 10^−5^	2.25 ± 0.03	0.61562 ± 3 × 10^−5^	0.01689 ± 1 × 10^−5^
propylene glycol	1.15131 ± 3 × 10^−5^	0.0082 ± 1 × 10^−4^	2.75 ± 0.02	0.87613 ± 3 × 10^−5^	0.01092 ± 8 × 10^−6^
glycerol	1.6062 ± 3 × 10^−5^	0.0622 ± 4 × 10^−4^	7.99 ± 0.05	0.53803 ± 3 × 10^−5^	0.0181 ± 1 × 10^−5^

